# Geometry Transfer Using Cross Sections for Sequential Mechanical–Electrostatic Simulation of Microstructured Flexible Capacitive Pressure Sensors

**DOI:** 10.3390/mi17101190

**Published:** 2026-10-14

**Authors:** Yiwei Shao, Wenzhe Bi, Qi Zhang, Xinyu Zhang, Xiaoya Liang, Kangping Xiao, Yulong Zhao

**Affiliations:** State Key Laboratory for Manufacturing Systems Engineering, School of Mechanical Engineering, Xi’an Jiaotong University, Xi’an 710049, China; imporeed@stu.xjtu.edu.cn (Y.S.); biwenzhe@stu.xjtu.edu.cn (W.B.); zhangxinyu0319@stu.xjtu.edu.cn (X.Z.); liang_xiaoya@stu.xjtu.edu.cn (X.L.); 3125301149@stu.xjtu.edu.cn (K.X.)

**Keywords:** capacitive pressure sensor, cross-sectional area, finite element analysis, geometry transfer, sequential mechanical–electrostatic simulation

## Abstract

Predicting how microstructure dimensions affect the response of flexible capacitive pressure sensors can guide sensor design. Large deformation and contact, however, complicate the construction of dielectric and air domains from the deformed mechanical geometry. This study presents a geometry transfer method based on equivalent cross sections. The method uses the finite element software packages Abaqus 2023 and COMSOL Multiphysics 6.2 for mechanical and electrostatic analysis, respectively. The electrostatic domains are reconstructed from horizontal section areas of the deformed boundary, current polydimethylsiloxane layer thicknesses and microstructure height. The method was evaluated using two square frustum geometries and measurements from three independent devices. Five equivalent square sections yielded volume meshes and capacitance solutions for all 29 selected states of each geometry. For the baseline geometry, capacitance differed from a 17-section reference by at most 0.1092% across four states spanning 0–411.85 kPa. Replacing equivalent squares with fitted contours of equal area changed capacitance by at most 0.04835%. A microscopy-guided geometry adjustment preserved the initial electrode separation and sidewall angle. Over 0–350 kPa, the adjustment reduced the root mean square error between the predicted relative capacitance response and the experimental mean from 0.0721 to 0.0292. The method provides a practical route from nonlinear deformation to capacitance prediction for microstructure design.

## 1. Introduction

Microstructured flexible capacitive pressure sensors combine high sensitivity with conformability [1,2,3,4], supporting wearable physiological monitoring, as well as electronic skin and robotic tactile sensing [5,6,7]. Their pressure response depends on the mechanical properties of the dielectric layer [4,8,9,10] and on microstructure shape, dimensions and spacing [11,12,13,14]. Compression changes electrode separation and the distribution of dielectric material and air as gaps narrow and contact develops [1,5,15,16]. Predicting capacitance from these geometric changes can aid in the selection of microstructure dimensions for a desired pressure response.

The compression of nearly incompressible polydimethylsiloxane (PDMS) involves material nonlinearity, large deformation and changing contact [17]. COMSOL Multiphysics can calculate both mechanical deformation and capacitance [18,19,20,21,22,23], but changes in contact can hinder convergence of the mechanical solution [24]. Here, Abaqus/Standard handles the nonlinear contact analysis, with hybrid solid elements suited to near incompressibility [25]. COMSOL Multiphysics then calculates the electric field and capacitance from the deformed configurations.

Previous studies have transferred deformation fields or deformed configurations from Abaqus to COMSOL Multiphysics for capacitance calculation [26,27,28]. The cited studies do not detail the exchange formats and geometric operations used for this transfer [26,27,28]. A mechanical mesh describes the deformed solid, whereas electrostatic analysis also requires surrounding air. The transfer must therefore turn the deformed solid boundary into closed dielectric and air domains suitable for volume meshing.

This study presents a geometry transfer method based on equivalent cross sections for simulations of microstructured capacitive pressure sensors. Horizontal sections of the deformed Abaqus boundary are replaced by squares of equal area. Together with the current PDMS layer thicknesses and microstructure height, these sections define reconstructed dielectric and air domains. With the mechanical states and layer dimensions held fixed, the section count and contour shape are varied to quantify their effects on capacitance. The method is evaluated using two square frustum geometries. One exhibits the baseline dimensions, and the other incorporates an adjustment guided by a scanning electron microscopy (SEM) image of an assembled cross section. Their predicted pressure responses are compared with measurements from three independent devices.

## 2. Materials and Methods

### 2.1. Geometry Transfer and Sequential Mechanical–Electrostatic Simulation

**Figure 1:**
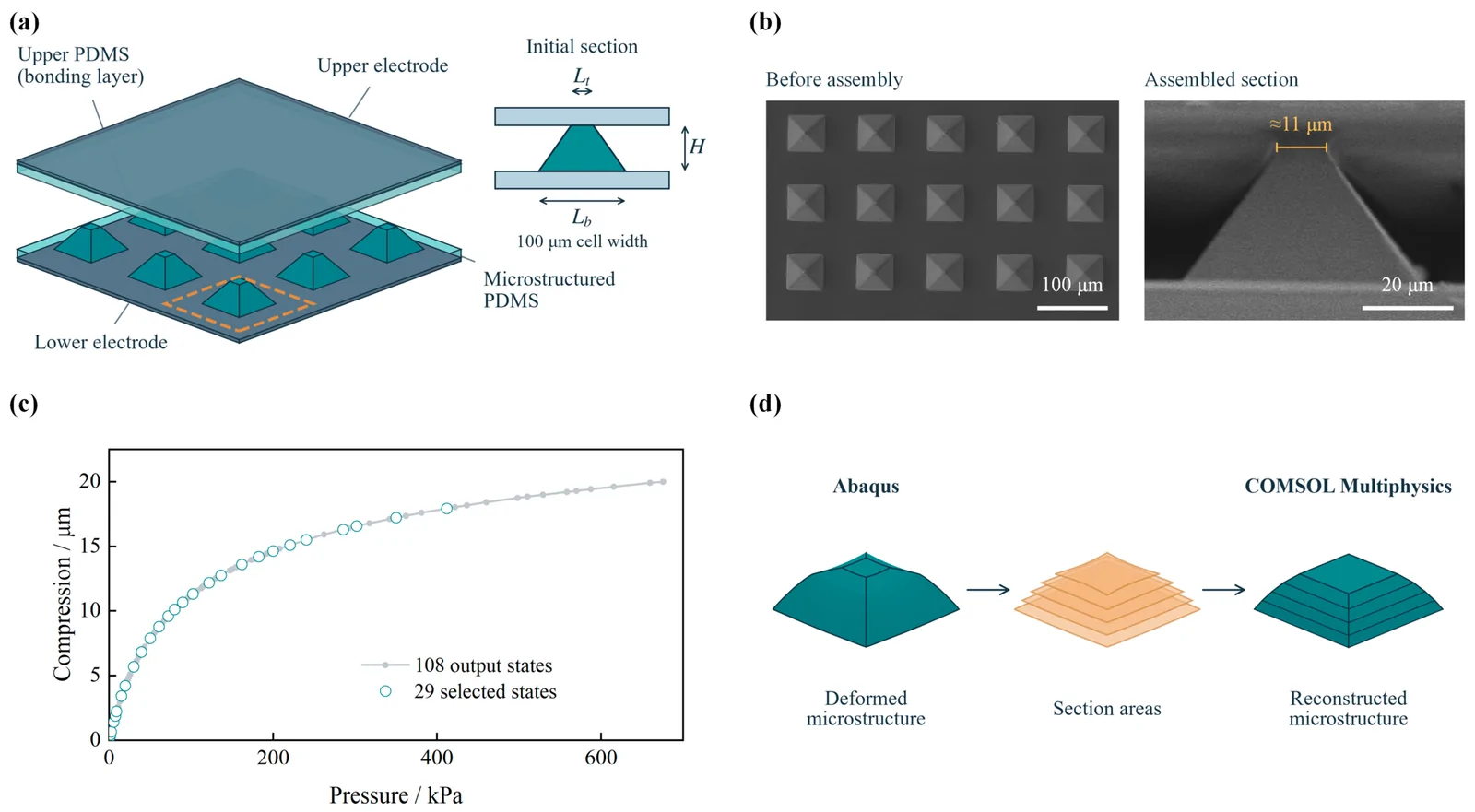
Sensor structure and transfer from mechanical states to electrostatic domains. (**a**) Exploded schematic of the periodic microstructured PDMS between the electrode surfaces. Upper PDMS is the cured bonding layer; the external protective layers are outside the modeled region. The dashed outline marks a unit cell with a side length of 100 μm. The initial section at right is drawn to scale using the baseline dimensions and defines bottom side length *L*_b_, top side length *L*_t_ and height *H*; dimensions are listed in Table 1. (**b**) SEM images of the replicated square pyramid array before assembly (**left**) and the assembled cross section (**right**). The microstructure meets the upper PDMS through a rounded bonding junction. The caliper marks an apparent neck width of approximately 11 μm immediately below this junction. Scale bars: 100 μm (**left**) and 20 μm (**right**). (**c**) Pressure and compression for 108 mechanical output states of the baseline geometry, with the 29 selected states marked by open circles. (**d**) Geometry transfer at 102.20 kPa, from the deformed microstructure in Abaqus through five horizontal section areas to the reconstruction in COMSOL Multiphysics. The PDMS layers are omitted from these views to clearly show the microstructure; both layers are included in the simulations.

The sensor contained a microstructured PDMS layer bonded to the upper electrode through a PDMS bonding layer, with protective PDMS outside both electrodes (Figure 1a,b). The modeled region comprised the cured bonding layer, termed upper PDMS, the microstructure, and the lower PDMS layer. The outer surfaces of the upper and lower layers defined the electrode boundaries. Because the bonding layer partly covered the microstructure tips after assembly, the assembled microstructure was represented by a square frustum with parallel square top and bottom faces. A square unit cell with a side length of 100 μm represented the periodic array.

The initial dimensions were based on the spin-coated PDMS thickness and nominal frustum geometry. The lower layer was 10.00 μm thick, and the baseline frustum height was 26.485 μm at a top side length *L*_t_ of 12.50 μm. The upper bonding layer was assigned a comparable nominal thickness of 10.015 μm, giving an initial electrode separation of 46.50 μm. A second geometry used *L*_t_ = 11.00 μm, based on the apparent neck width immediately below the rounded upper junction in the calibrated SEM cross section (Figure 1b). For this smaller top side length, preserving the sidewall angle and initial electrode separation required a greater microstructure height *H* and a smaller upper PDMS layer thickness *t*_u_ (Table 1). Both geometries used the same material parameters, contact conditions and reconstruction procedure.

A third-order Yeoh model described the PDMS response [29]. Because PDMS stiffness depends on curing and test conditions [30], the model was calibrated against tensile measurements on material prepared for this study. Uniaxial tensile testing, commonly used for PDMS hyperelastic calibration [31,32,33], was performed on three independently prepared specimens. The specimens were prepared from Sylgard 184 PDMS at a 5:1 mass ratio of base to curing agent and cured at 70 °C for 3 h. Their nominal stress–strain curves were interpolated at common engineering strains to obtain the mean and sample standard deviation (SD, *n* = 3).

**Figure 2:**
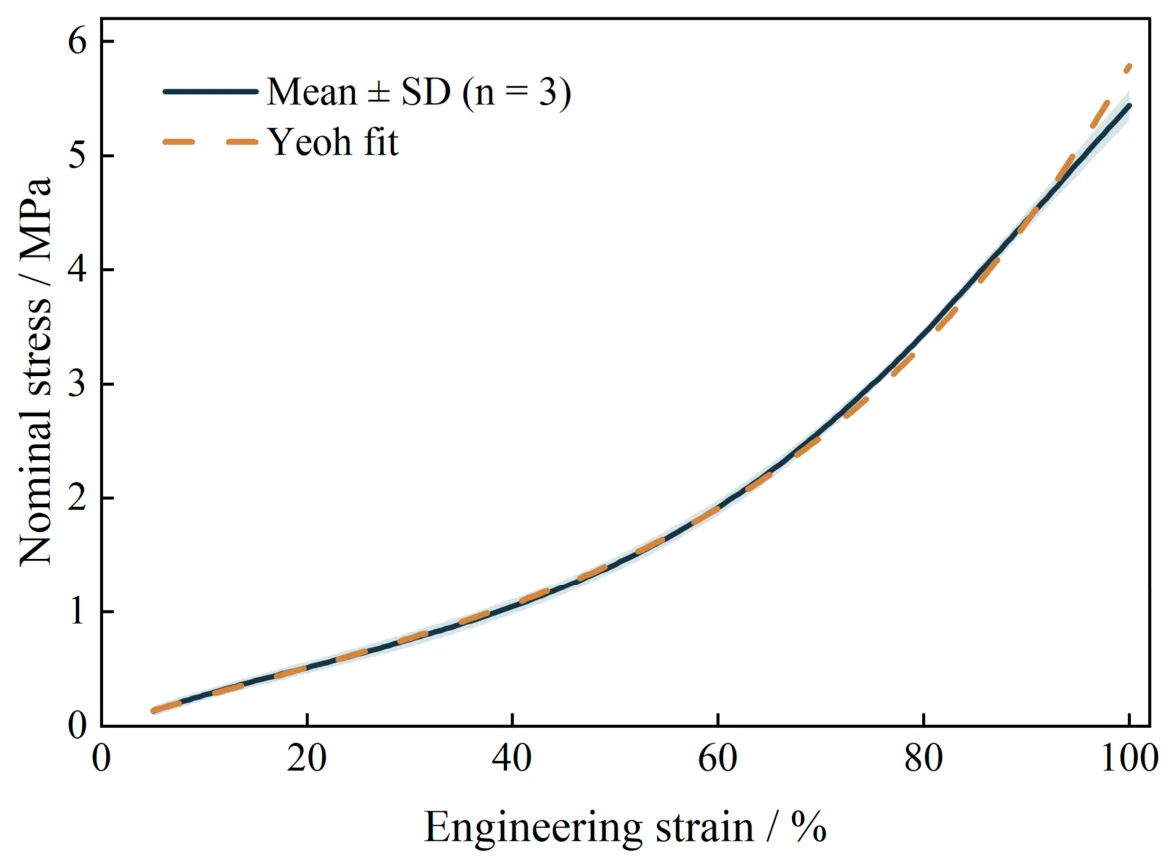
PDMS tensile characterization and Yeoh model calibration. Mean nominal stress–strain curve for three independently prepared 5:1 PDMS specimens and the Yeoh fit over a 5–100% engineering strain. The shaded band denotes ±1 sample standard deviation.

Fitting the mean curve over a 5–100% engineering strain gave *C*_10_ = 0.476 MPa, *C*_20_ = 0.126 MPa and *C*_30_ = 0.0560 MPa (Figure 2). A Poisson ratio of 0.49 was adopted as a near-incompressible approximation for the initial PDMS response, following previous modeling studies [34,35]. Hybrid elements were used to accommodate this material behavior [25].

Abaqus/Standard 2023 calculated quasi-static compression along *y* in a quarter cell initially spanning 0–50 μm in *x* and *z*. The model included geometric nonlinearity and used 82,496 C3D8RH hybrid hexahedral elements with reduced integration. Displacement normal to each lateral face was constrained at *x* = 0 and 50 μm and at *z* = 0 and 50 μm. Shared nodes connected the microstructure continuously to both PDMS layers. The lower outer surface was fixed, and the upper outer surface moved in the negative *y* direction, with its *x* and *z* displacements constrained. Self-contact was defined on the inner PDMS layer surfaces and microstructure sidewalls that initially faced air. Finite sliding surface-to-surface contact was used, with hard normal contact and Coulomb friction (*μ* = 0.3). Compression, nodal displacements and total reaction force on the loading face were recorded at up to 20 μm compression, giving 108 output states for the baseline geometry and 109 for the 11 μm geometry.

Pressure was calculated as *p* = |*F*_R_|/*A*_rep_, where *F*_R_ is the total reaction force, and *A*_rep_ = 0.0025 mm^2^ is the quarter cell area. For each geometry, the output state closest to each of 28 target pressures was selected. Including the initial state, this yielded 29 states for section extraction, each retaining its calculated pressure; Figure 1c shows the baseline selection. The target pressures were 0.5, 1, 2, 3, 5, 7.5, 10, 15, 20, 30, 40, 50, 60, 70, 80, 90, 100, 120, 140, 160, 180, 200, 225, 250, 275, 300, 350 and 400 kPa.

**Figure 3:**
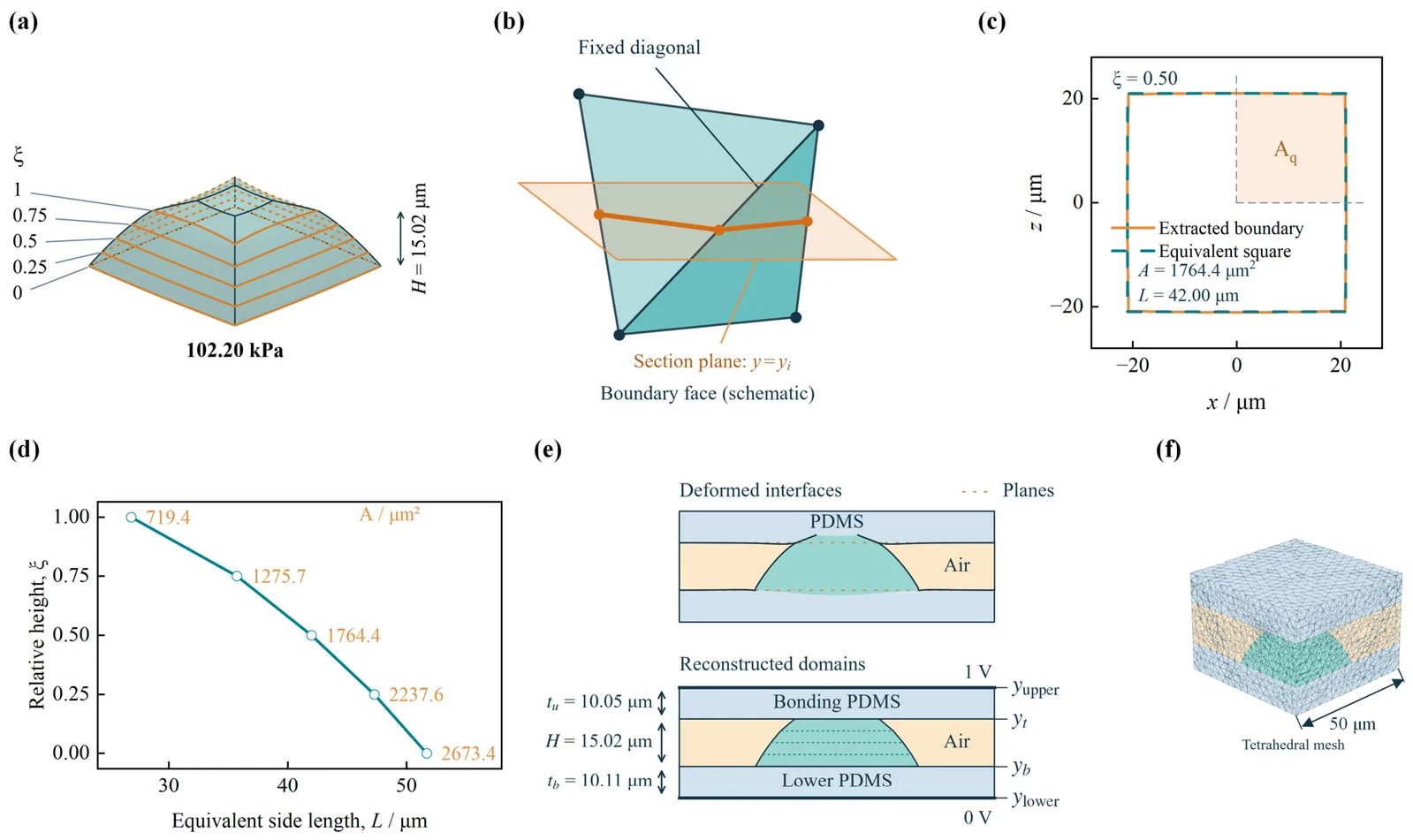
Geometry reconstruction from exterior boundary intersections for the baseline geometry at 102.20 kPa. (**a**) Full deformed microstructure displayed using model symmetry, with five horizontal section contours at relative heights *ξ* = 0, 0.25, 0.50, 0.75 and 1, measured between the representative inner planes *y*_b_ and *y*_t_. Rear portions of the section contours are dashed. (**b**) Schematic (not to scale) of the intersection of a warped face, divided along a fixed diagonal into two triangles, with the section plane *y* = *y_i_*. (**c**) Full mid-height section obtained by symmetry and its equivalent square. Shading marks the quarter section area *A*_q_; the full area is *A* = 4*A*_q_ = 1764.4 μm^2^, and the equivalent side is $L=\sqrt{A}=42.00$ μm. (**d**) Equivalent side length through the current height; orange values give the full section areas. (**e**) Deformed interfaces in the central plane *z* = 0 (**above**) and reconstructed PDMS and air domains (**below**), shown at the same scale. The four labeled heights define the electrode planes and representative inner planes. Orange dashed lines mark the inner planes. The reconstructed lower and upper PDMS layers are 10.11 and 10.05 μm thick, respectively, and the microstructure is 15.02 μm high. Dashed divisions show the four frustum segments before unification; electrode potentials are indicated. (**f**) Quarter cutaway of the full-cell tetrahedral mesh, with domain colors matching (**e**).

Each selected state supplied the geometry for electrostatic reconstruction in COMSOL Multiphysics 6.2 (Figure 1d), producing a full-cell model (Figure 3e,f). The calculation was sequential, with no feedback of electrostatic forces to the mechanical model. The current coordinates were ***x*** = ***X*** + ***U***, where ***X*** and ***U*** denote initial coordinates and displacement. The outer PDMS surfaces remained horizontal under the prescribed boundary conditions. Each inner surface was represented by a horizontal plane through a reference node initially located at *x* = 50 μm and *z* = 0, outside the microstructure footprint. This location defined the layer height away from the local curvature near the microstructure junction. Outer-surface nodes at the same lateral location defined the electrode planes.

The four plane heights, from bottom to top, were *y*_lower_, *y*_b_, *y*_t_ and *y*_upper_ (Figure 3e). They defined the current layer thicknesses, *t*_b_ = *y*_b_ − *y*_lower_ and *t*_u_ = *y*_upper_ − *y*_t_, and the microstructure height, *H* = *y*_t_ − *y*_b_. Five horizontal sections were taken at *y_i_* = *y*_b_ + *ξ_i_H*, with relative heights *ξ_i_* = 0, 0.25, 0.50, 0.75 and 1.00 (Figure 3a). The end sections defined the bottom and top dimensions, while the three interior sections captured changes in width through the height.

The microstructure boundary was identified by removing faces shared by elements within the microstructure region. It comprised the side faces, symmetry cut faces and interfaces with the PDMS layers, with connectivity fixed during loading. Each quadrilateral face was divided along a fixed diagonal into two triangles, giving a piecewise planar representation for section intersection when its four vertices were not coplanar (Figure 3b).

The triangles were intersected with each horizontal plane, and the resulting segments were joined into a closed contour in the *x*–*z* plane. With contour vertices (*x_j_*, *z_j_*) ordered counterclockwise, the quarter section area was calculated using Equation (1),

(1) $$A_{q,i}=\frac{1}{2}\sum_{j}(x_{j}z_{j+1}-x_{j+1}z_{j}),$$

with cyclic vertex indices. Nodes within 2 × 10^−12^ mm of a section plane were treated as coplanar, and endpoints within 2 × 10^−11^ mm were merged. For boundary patches coplanar with a section plane, the contour was obtained from adjacent noncoplanar faces.

Symmetry gave the full section area *A_i_* = 4*A*_q,*i*_ and equivalent square side length $L_{i}=2\sqrt{A_{q,i}}$ (Figure 3c,d). Five squares were centered on the cell axis, with sides parallel to *x* and *z*. Four frustum segments joined adjacent squares into a single microstructure domain, with side length varying linearly through each segment. PDMS layers of current thicknesses *t*_b_ and *t*_u_ covered the full cell below and above the microstructure, and air filled the remaining volume between their inner planes (Figure 3e).

The relative permittivity of PDMS was set to 2.75 using the COMSOL Multiphysics material library as a nominal material parameter. This value has also been used in the finite element modeling of a PDMS pressure sensor [36]. The relative permittivity of air was 1, and these values were held constant across the reconstructed geometries. The upper electrode was held at 1 V and the lower grounded; a zero-charge condition was applied to the remaining exterior boundaries.

A free tetrahedral mesh was generated independently for each reconstructed state (Figure 3f). Automated meshing accommodated the changes in PDMS and air geometry between states. Each electrostatic solution used a fixed mesh. The standard setting used maximum and minimum element size controls of 3.5 and 0.15 μm, respectively, a maximum growth rate of 1.35, a curvature factor of 0.3 and a narrow-region resolution of 0.85. The effect of mesh refinement on the calculated capacitance was assessed using the finer setting described in Section 2.2.

The electrostatic solution gave the full cell capacitance *C*_cell_. Array capacitance was calculated as *C* = *C*_cell_*A*_e_/*A*_cell_, with electrode area *A*_e_ = 56.25 mm^2^, and cell area *A*_cell_ = 0.01 mm^2^. Using the calculated unloaded capacitance *C*_0_ of each geometry, the relative response was defined as Δ*C*/*C*_0_ = [*C*(*p*) − *C*_0_]/*C*_0_.

### 2.2. Numerical Verification

Two electrostatic comparisons evaluated axial sampling and the equivalent square approximation for the baseline geometry (*L*_t_ = 12.50 μm). Both used the initial state at 0 kPa and three compressed states at 9.20, 80.08 and 411.85 kPa. Within each state, the current PDMS layer thicknesses, microstructure height, material parameters and electrostatic boundary conditions were held fixed.

The axial comparison used *N* = 3, 5, 9 and 17 sections, with the spacing halved successively to give nested sampling heights. Each geometry was defined by its *N* sampled sections, with the equivalent square side length varying linearly between adjacent sections. Five sections thus defined four frustum segments, as in Section 2.1. The 17-section reconstruction was the finest directly sampled representation in this comparison and served as the numerical reference.

Relative capacitance and volume differences were expressed as percentages, 100|*C_N_* − *C*_17_|/*C*_17_ and 100|*V_N_* − *V*_17_|/*V*_17_, respectively. The absolute difference in Δ*C*/*C*_0_ between each *N*-section reconstruction and the 17-section reference was also evaluated. Here, *C_N_* and *V_N_* denote capacitance and reconstructed microstructure volume. Each section set used its own unloaded capacitance *C*_0,*N*_ for normalization. The volume was calculated from the constructed segments using Equation (2),

(2) $$V_{N}=\sum_{i}\frac{y_{i+1}-y_{i}}{3}(L_{i}^{2}+L_{i}L_{i+1}+L_{i+1}^{2}).$$

The contour comparison retained the five section areas and heights. P4 denoted the equivalent square; P16 and P64 denoted polygons with 16 and 64 vertices sampled from a fitted contour. Radial sampling converted the extracted contour coordinates into a common angular representation for fitting and for establishing corresponding vertices at adjacent heights. In the positive *x*–*z* quadrant, 361 rays were cast from the section center at 0.25° intervals, with *θ* measured from the *x*-axis. The distance to the outer contour defined the radius at each angle. Radii at *θ* and π/2−*θ* were averaged to enforce symmetry under exchange of *x* and *z*. The radial profile in Equation (3) was fitted by least squares,

(3) $$r_{i}(\theta )=b_{i}/\max(|\cos\theta |,|\sin\theta |)+\sum_{k=0}^{6}c_{\mathrm{ik}}\cos(4\mathrm{k\theta}),$$

where *b_i_* = *L_i_*/2 and the *c_ik_* are fitted coefficients. The fitted curve was rescaled to the area obtained from boundary intersections. Uniform angular sampling produced P16 and P64, each rescaled to the P4 area so that section shape could be compared at the fixed area. Corresponding vertices at adjacent heights were joined by triangulated side faces. For each section, P64 and the extracted contour were compared at the same 361 angles. The radius difference at each angle was divided by the extracted radius and expressed as a percentage; the root mean square (RMS) and maximum absolute values quantified contour fidelity.

P64 served as the numerical reference for the contour comparison. The percentage difference 100|*C*_P4_ − *C*_P64_|/*C*_P64_ quantified the effect of the square approximation. The corresponding percentage difference between P16 and P64, 100|*C*_P16_ − *C*_P64_|/*C*_P64_, quantified the sensitivity to the number of contour vertices.

Electrostatic mesh comparisons estimated numerical noise by refining the standard mesh from automatic level 2 to level 1. The finer setting used maximum and minimum element size controls of 2.0 and 0.02 μm, respectively, a maximum growth rate of 1.30, and a curvature factor of 0.2. For the five-section square geometry at 411.85 kPa, the element count increased from 129,071 to 624,305. The comparisons covered this geometry at 0, 80.08 and 411.85 kPa, and P16 and P64 at 411.85 kPa. Relative differences used level 1 capacitance as the denominator for the square geometry and level 2 capacitance for P16 and P64.

At the same four states used for the axial and contour comparisons, the inner PDMS surfaces initially facing air were compared with their representative planes. The area-weighted RMS height deviation was calculated using current surface areas on the triangulated boundary.

Mesh generation was checked for all 29 reconstructed states of each geometry. A direct-import test at 102.20 kPa used the complete quarter cell volume mesh of the baseline geometry exported from Abaqus in NASTRAN format. The mesh was imported into the COMSOL Multiphysics geometry sequence, with simplification disabled. Its free tetrahedral meshing outcome was compared with that of the five-section reconstruction at the same state (Section 3.1).

### 2.3. Device Fabrication and Testing

Sylgard 184 PDMS was prepared at a 5:1 mass ratio of base to curing agent and spin-coated on silicon molds. Curing at 70 °C for 3 h produced PDMS layers with square pyramid microstructures. Silver electrodes were screen-printed on the back of the microstructured layer and on a separate PDMS protective layer, then cured at 90 °C for 30 min.

The bonding solution contained 12.5 wt% of the same PDMS prepolymer mixture in n-hexane and was spin-coated onto the upper electrode. After partial curing at 50 °C for 30 min, the upper and lower assemblies were joined, with the bonding layer partly covering the microstructure tips. The devices were then cured at 70 °C for 3 h.

**Figure 4:**
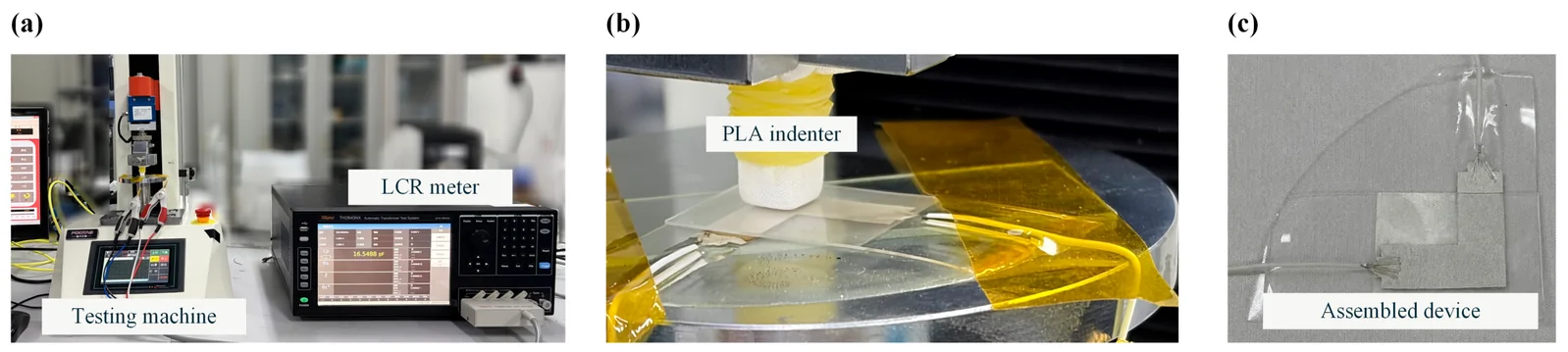
Experimental setup and assembled device. (**a**) Testing machine and LCR meter. (**b**) Loading contact with the polylactic acid (PLA) indenter. (**c**) Assembled device. Capacitance was recorded at 100 kHz and 1 V during displacement-controlled loading.

Each of three independent devices underwent three loading–unloading runs under displacement control using the setup in Figure 4a. A polylactic acid (PLA) indenter (Figure 4b) applied the load to the assembled device (Figure 4c). Each protective PDMS layer outside the electrodes was approximately 500 μm thick. The testing-machine displacement included deformation of the protective layers outside the electrodes and therefore, differed from the compression of the modeled region between the electrodes. Pressure was calculated from the measured force divided by the loaded area of 56.25 mm^2^. An LCR meter recorded capacitance in parallel-capacitance (Cp) mode at 100 kHz and 1 V.

After time alignment, force and capacitance were averaged over a 5 s window ending 2 s before the end of each displacement hold. For each run, the unloaded capacitance *C*_0_ was the mean capacitance over the longest available window, up to 5 s, within the initial unloaded interval. The relative capacitance response was calculated using that run’s *C*_0_. Pressure and relative response at corresponding loading stages were averaged across the three runs for each device. The three device curves were then linearly interpolated at common pressures to calculate the mean and sample SD across devices (*n* = 3). For the group summary of *C*_0_, the three run values were first averaged within each device, followed by the mean and sample SD across devices.

Experimental sensitivity was estimated separately for each device over 0–50, 50–150 and 150–350 kPa using the loading curve averaged over its three runs. A least-squares line was fitted to Δ*C*/*C*_0_ versus pressure using the measured stage values and linearly interpolated response values at the interval endpoints. Sensitivity was defined as the slope of this fit, reported as the mean and sample SD across the three devices.

The simulated loading response of each geometry was compared with the experimental mean over 0–350 kPa, an interval covered by all three devices and both simulations. Piecewise linear interpolation of the simulated and experimental Δ*C*/*C*_0_ curves gave responses at common pressures, denoted as *η*_sim_(*p*) and *η*_exp_(*p*). The signed difference was *η*_sim_(*p*) − *η*_exp_(*p*). Equation (4) gives the root mean square error (RMSE), averaged over pressure,

(4) $$\mathrm{RMSE}=[\frac{1}{p_{\max}}\int_{0}^{p_{\max}}(\eta_{\mathrm{sim}}(p)-\eta_{\exp}(p){)}^{2}dp{]}^{1/2},$$

where *p*_max_ = 350 kPa.

## 3. Results and Discussion

### 3.1. Numerical Validity of the Geometry Transfer Method

The five-section reconstruction yielded closed section contours, volume meshes and capacitance solutions for all 29 selected states of the baseline geometry, reaching a compression of 17.93 μm at 411.85 kPa. The same procedure produced volume meshes and capacitance solutions for all 29 states of the geometry, with *L*_t_ = 11 μm, up to 412.47 kPa. At 102.20 kPa, direct import of the complete deformed volume mesh of the baseline geometry produced a domain in COMSOL Multiphysics, but self-intersecting faces prevented free tetrahedral meshing. Reconstruction from five sections at the same state yielded a volume mesh and a capacitance solution (Figure 3f).

At 102.20 kPa, the reconstructed microstructure height was 15.02 μm, compared with an initial height of 26.49 μm (Figure 3a,c–e). The equivalent side lengths were 51.70 μm at the bottom, 42.00 μm at mid-height and 26.82 μm at the top. Relative to the initial geometry shown in Table 1, the microstructure was shorter and wider, with greater lateral expansion near the upper interface. Its equivalent side length no longer varied linearly with relative height (Figure 3d). The intermediate sections capture this variation through successive frustum segments.

**Figure 5:**
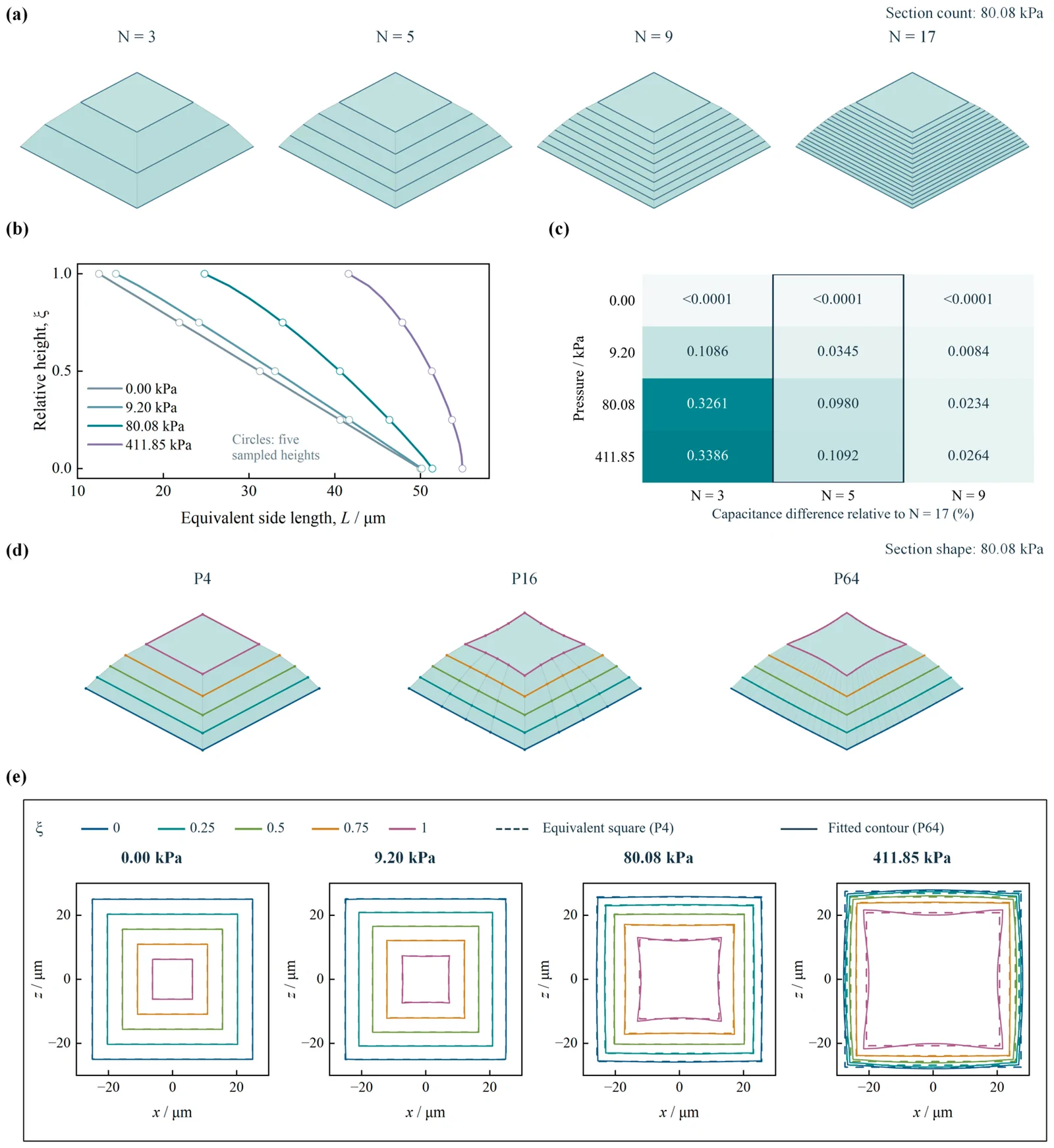
Numerical comparisons for the baseline square frustum (*L*_t_ = 12.50 μm). (**a**) Microstructures reconstructed with *N* = 3, 5, 9 and 17 sections at 80.08 kPa; adjacent sections form *N* − 1 frustum segments. (**b**) Equivalent side length profiles from 17 sections at 0, 9.20, 80.08 and 411.85 kPa; circles mark the five nested section positions. (**c**) Relative capacitance differences for *N* = 3, 5 and 9 against *N* = 17 at the same four states. Cell labels give percentages, darker shading indicates larger capacitance differences. Initial-state differences are below 0.0001%. (**d**) P4, P16 and P64 microstructure reconstructions at 80.08 kPa, with the same five section heights and areas. The model views in (**a**,**d**) use the same viewing angle and geometric scale. (**e**) Concentric P4/P64 section outlines at the four states, all at the same geometric scale. Colors in (**d**,**e**) identify relative height, *ξ*. In (**e**), dashed lines denote equivalent squares (P4), and solid lines denote fitted contours (P64); each pair has the same area.

Figure 5a compares the microstructures reconstructed with 3, 5, 9 and 17 sections at 80.08 kPa. The 17-section profiles show how the equivalent side length varied with height at 0, 9.20, 80.08 and 411.85 kPa (Figure 5b). Across these four states, the maximum capacitance difference relative to the 17-section reference decreased from 0.3386% for three sections to 0.1092% for five and 0.02636% for nine (Figure 5c). With five sections, the maximum absolute difference in Δ*C*/*C*_0_ was 0.002447, and the maximum relative volume difference was 0.7915%. All three maxima for five sections occurred at 411.85 kPa. The volume comparison covered only the reconstructed microstructure, whereas capacitance depends on the distribution of PDMS and air throughout the stack [5,37,38]. The five-section reconstruction, therefore, gave capacitance predictions close to those of the 17-section reference, while reducing section extraction and reconstruction work. Each reconstructed state required only four frustum segments.

Among the 20 sections from the four states, the largest RMS relative radial deviation of P64 from the extracted contour was 0.2173%, and the largest absolute relative deviation among the sampled angles was 1.8714%. The fitted contours departed visibly from the equivalent squares at some heights (Figure 5d,e). Relative to P64, the maximum capacitance difference was 0.04835% for P4 and 0.02596% for P16, both at 80.08 kPa (Table 2). Changing the contour while keeping its area fixed changes the lateral distribution of PDMS and air, which can affect the electric field and capacitance. In the four tested states, the equivalent squares gave capacitances close to those of the fitted-contour models.

At 411.85 kPa, the capacitance difference between P4 and P64 was 0.04732%. Mesh refinement of P16 and P64 at the same pressure produced smaller changes of 0.004552% and 0.005120%, respectively. For the five-section square model, the maximum change on refinement was 0.003250% across 0, 80.08 and 411.85 kPa.

### 3.2. Experimental Comparison

**Figure 6:**
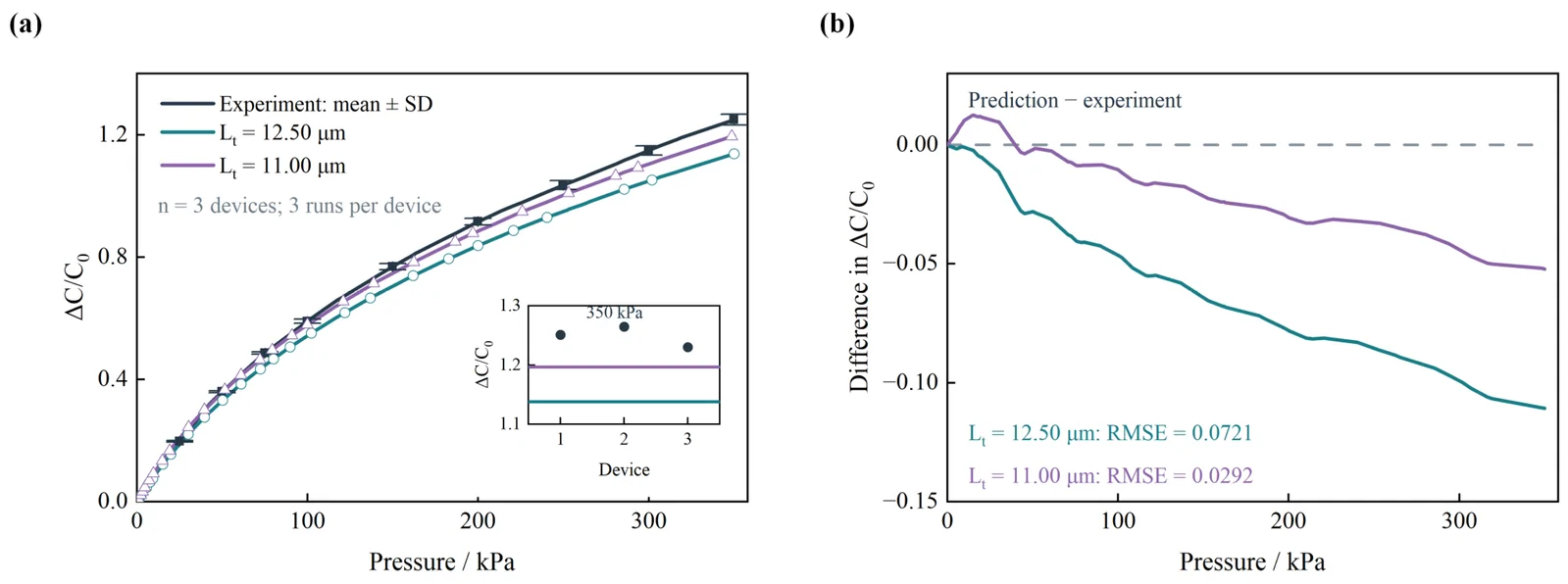
Experimental responses and predictions for two square frustum geometries. (**a**) Predicted responses for *L*_t_ = 12.50 and 11.00 μm and the experimental mean over 0–350 kPa. Both geometries have the same initial electrode separation and sidewall angle. The dark line shows the mean of three independent device responses, with each device response first averaged over three loading runs. Squares at common reporting pressures show the experimental mean, with error bars indicating sample SD across devices. Open circles and open triangles mark the calculated states for *L*_t_ = 12.50 and 11.00 μm, respectively. The inset shows Δ*C*/*C*_0_ for each device at 350 kPa and both predicted values as horizontal lines. (**b**) Prediction minus experimental mean for each geometry; the dashed line marks zero. RMSE is evaluated over pressure. Both simulations and the experimental runs use their own unloaded capacitances for normalization.

The three devices had an unloaded capacitance of 16.6120 ± 0.0860 pF (mean ± sample SD, *n* = 3), after averaging the three runs within each device. Their relative capacitance response increased with pressure (Figure 6a), while the interval sensitivity decreased (Table 3).

A similar decrease in sensitivity was reported for Shi et al.’s capacitive sensor [9]. Merces et al. and Xu et al. reported normalized current responses over different pressure intervals [39,40]. Current increases with junction contact area in the device reported by Merces et al. and with decreasing contact resistance in Xu et al.’s sensor.

The baseline model predicted an unloaded capacitance of 16.2548 pF and a relative response below the experimental mean at nonzero pressures up to 350 kPa (Figure 6a,b). Its RMSE over 0–350 kPa was 0.0721. At 350 kPa, the predicted Δ*C*/*C*_0_ was 1.1377, compared with the experimental value of 1.2485 ± 0.0173 (mean ± sample SD, *n* = 3). The difference of 0.1108 amounted to 8.88% of the experimental mean.

The SEM image of the assembled cross section showed an apparent neck width of approximately 11 μm immediately below the upper junction (Figure 1b), compared with the baseline top side length of 12.50 μm. Reducing *L*_t_ to 11 μm, while adjusting *H* and *t*_u_ to preserve the initial electrode separation and sidewall angle (Table 1), increased compression at 350 kPa from 17.2187 to 17.9272 μm. The predicted Δ*C*/*C*_0_ increased to 1.1964, reducing the underprediction at that pressure from 8.88% to 4.18% (Figure 6a,b). Over 0–350 kPa, the RMSE fell from 0.0721 to 0.0292, a reduction of 59.54%.

Agreement in Δ*C*/*C*_0_ improved mainly at intermediate and higher pressures. At 25 kPa, the prediction for *L*_t_ = 11 μm exceeded the experimental mean by 5.39%, whereas the baseline prediction was 4.25% lower. For the 11 μm geometry, the calculated *C*_0_ was 15.9160 pF, further below the measured value. Although the initial electrode separation was unchanged, the adjustment altered the distribution of PDMS and air and hence, *C*_0_. At 350 kPa, the calculated capacitance increment Δ*C* increased from 18.4932 to 19.0413 pF. The larger increment and lower *C*_0_ both increased the normalized response. The geometric adjustment therefore improved agreement in Δ*C*/*C*_0_ over the comparison interval while increasing the discrepancy in unloaded capacitance.

Rounded bonding fillets at the upper junction (Figure 1b) may alter lateral restraint and local compression relative to the idealized frustum. During geometry transfer, a separate approximation replaces the deformed inner PDMS surfaces with horizontal planes (Figure 3e). The upper-surface RMS height deviation increased from 0.2951 μm at 80.08 kPa to 0.7831 μm at 411.85 kPa. At 411.85 kPa, the lower-surface RMS deviation was 0.1552 μm, and the maximum absolute deviations were 0.3705 μm for the lower surface and 3.8021 μm for the upper surface. Both the unmodeled bonding fillets and this planar approximation may contribute to the pressure-response discrepancy.

The Yeoh model was calibrated against the mean uniaxial tensile curve, yielding a fit with *R*^2^ = 0.9979. In the sensor, however, the microstructure underwent constrained compression and contact. Hyperelastic predictions can lose accuracy when the deformation mode differs from the calibration test [41,42]. In comparisons using rubber datasets, the Yeoh model described large uniaxial deformation well but showed larger errors under biaxial loading [43]. If the reaction force for a given deformed geometry were overestimated, its capacitance would be assigned to a higher pressure. This would shift the predicted response to the right and could contribute to the remaining underprediction at higher pressures.

The relative permittivity of the 5:1 PDMS prepared using the device curing schedule has not been measured at 100 kHz. The nominal value of 2.75 is therefore a source of uncertainty in the capacitance prediction and may contribute to the remaining discrepancy in the experiment.

Comparing the predicted relative capacitance change and sensitivity across microstructure dimensions can guide prototype selection for a target pressure interval. Numerical screening before fabrication could reduce repeated material preparation, device assembly and performance testing, saving development time and prototype costs.

## 4. Conclusions

This study developed a geometry transfer method based on equivalent cross sections to connect nonlinear mechanical analysis with capacitance prediction in microstructured capacitive pressure sensors. Current PDMS layer thicknesses, microstructure height and horizontal section areas define meshable dielectric and air domains. In the two square frustum geometries used for verification, five equivalent square sections produced volume meshes and capacitance solutions for all 29 selected states of each geometry. At four baseline states spanning 0–411.85 kPa, the maximum capacitance difference from the 17-section reference was 0.1092%, and replacing squares with fitted contours of equal area changed capacitance by at most 0.04835%. With the layer planes held fixed, these comparisons support five equivalent square sections as a compact representation of the deformed microstructure for capacitance calculation.

Comparison with measurements from three independent devices showed how the method can assess the effects of geometric changes on the predicted pressure response. A microscopy-guided adjustment preserved the initial electrode separation and sidewall angle. It reduced the RMSE between the predicted relative capacitance response and the experimental mean from 0.0721 to 0.0292 over 0–350 kPa. Reconstruction using section geometry avoids transferring the complete deformed volume mesh while retaining the dimensions needed to define the dielectric and air domains. This provides a practical basis for comparing microstructure dimensions through their predicted pressure responses and selecting candidate geometries before fabrication.

## Figures and Tables

**Table 1:** Initial dimensions of the two square frustum geometries.

Top Side, *L*_t_/μm	Height, *H*/μm	Upper PDMS Thickness, *t*_u_/μm
12.50	26.485	10.015
11.00	27.541	8.959

Both geometries used a bottom side length of 50.00 μm, a unit cell side length of 100 μm and a lower PDMS thickness of 10.00 μm. The sum of *H* + *t*_u_ was 36.50 μm, giving an initial electrode separation of 46.50 μm.

**Table 2:** Relative capacitance differences for the baseline contour comparison (*L*_t_ = 12.50 μm).

Pressure/kPa	P4 Versus P64/%	P16 Versus P64/%
0.00	<0.0001	<0.0001
9.20	0.0005	0.0008
80.08	0.0483	0.0260
411.85	0.0473	0.0068

Differences are expressed as percentages, 100|*C*_P*n*_ − *C*_P64_|/*C*_P64_, for *n* = 4 and 16. At each pressure, the five section areas, their heights and the current PDMS layer thicknesses are identical across the three representations.

**Table 3:** Experimental sensitivity and pressure ranges of selected pressure sensors.

Study	Sensing Mechanism and Structure	Normalized Output	Sensitivity/kPa^−1^	Pressure/kPa
Merces et al. [39]	Variable-area transport junction: metallic nanomembrane–molecular contact	Δ*I*/*I*_0_	480 (mean)	10^−5^–10^−2^ (detection range)
Shi et al. [9]	Capacitive: cone microstructures in a CB/CNT/PDMS dielectric	Δ*C*/*C*_0_	0.00397; 0.000736	0–600; 600–1300
Xu et al. [40]	Piezoresistive: contact resistance of hierarchical MWCNT/PDMS microdomes	Δ*I*/*I*_0_	0.705	0–200
This study	Capacitive: square frustum PDMS dielectric	Δ*C*/*C*_0_	0.00721 ± 0.00008; 0.00407 ± 0.00007; 0.00241 ± 0.00004	0–50; 50–150; 150–350

Multiple sensitivity values correspond to the pressure intervals in the listed order. Values for this study are the mean ± sample SD of slopes fitted separately to three independent device responses. For Merces et al., sensitivity is defined as (Δ*I*/*I*_0_)/Δ*p*; 480 kPa^−1^ is the reported mean, and the pressure column gives the detection range reported in their Table 1 rather than a linear-fit interval. The Xu et al. entry is the measured sensitivity of the sensor in their Figure 3a, with output expressed as Δ*I*/*I*_0_. CB, carbon black; CNT, carbon nanotube; MWCNT, multiwalled carbon nanotube.

## Data Availability

The original contributions presented in this study are included in the article. Further inquiries can be directed to the corresponding authors.
